# Uplink Scheduling of Navigation Constellation Based on Immune Genetic Algorithm

**DOI:** 10.1371/journal.pone.0164730

**Published:** 2016-10-13

**Authors:** Yinyin Tang, Yueke Wang, Jianyun Chen, Xianbin Li

**Affiliations:** 1 College of Mechatronics Engineering and Automation, National University of Defense Technology, Changsha, Hunan, China; 2 Troop of 69222, Luntai, Xinjiang, China; West Virginia University, UNITED STATES

## Abstract

The uplink of navigation data as satellite ephemeris is a complex satellite range scheduling problem. Large–scale optimal problems cannot be tackled using traditional heuristic methods, and the efficiency of standard genetic algorithm is unsatisfactory. We propose a multi-objective immune genetic algorithm (IGA) for uplink scheduling of navigation constellation. The method focuses on balance traffic and maximum task objects based on satellite-ground index encoding method, individual diversity evaluation and memory library. Numerical results show that the multi–hierarchical encoding method can improve the computation efficiency, the fuzzy deviation toleration method can speed up convergence, and the method can achieve the balance target with a negligible loss in task number (approximately 2.98%). The proposed algorithm is a general method and thus can be used in similar problems.

## 1 Introduction

Navigation constellations such as GPS, GLONASS, GALILEO, and BeiDou are the most commonly used global navigation satellite systems (GNSS). Navigation data including satellite ephemeris, clock error, satellite almanac, and ionosphere parameter need to be uplinked to the navigation satellite through the ground station so as to improve the accuracy of positioning, velocity, and timing (PVT) [1, 2]. The information between stations can be exchanged through wireless communication systems [3] or fiber–optical communication systems [4–6]. The physical impairments in fiber–optical communication systems are modeled and analyzed in [7–9]. With the development of navigation technology, establishing an inter–satellite link (ISL) in navigation constellations has become a trend [10–12]. Therefore, the information related to ISLs will be uplinked in the future.

Choosing the apt station to uplink a particular satellite is the so–called multi–resource range scheduling problem (MuRRSP). Many researchers aim to find a solution to this problem. An improved genetic algorithm using a station ID encoding method is proposed in [13]. The priority constraint is taken into account so that the important task is more likely to appear in the offspring. A Lagrangian heuristic method is used to tackle the large–scale practical problem in [14]. A two–phase heuristic algorithm based on a multi–objective mix–integer model is presented to uplink navigation data in [15]. Several standard meta–heuristics algorithms are applied to image downlink for synthetic aperture radar (SAR), and simulated annealing is found as the most efficient one [16]. A comparison of genetic, heuristic and local search algorithm is done in [17], and it is concluded that the genetic algorithm yields the best overall performance on larger, more difficult problems. The genetic algorithm is also applied for the satellite scheduling problem in [18, 19].

As mentioned above, heuristics and genetic algorithms are the most widely used methods in MuRRSP. Heuristics is suitable for simpler problems and is designed for specific problems, while the genetic algorithm can deal with complicated problems, and more importantly, it is a general solution for a series of problems. Hence, genetic algorithms have great potential in the satellite scheduling field. However, premature convergence is one drawback in the standard genetic algorithm (SGA) [20]. The immune genetic algorithm (IGA) is insensitive to premature convergence since the genetic probability is decided by not only the fitness but also the concentration [21–23]. The IGA is successfully used in many applications. In this paper, the IGA is chosen to solve the uplink problem in navigation constellation.

Researchers mainly consider the maximum task or maximum weight or maximum priority as the targets, but seldom consider the traffic balanced between the stations. In reality, building a ground station is expensive and must be done under many strict constraints such as visibility. Keep the load balance may bring some benefits [24]. If the traffic is concentrated on a few stations, then the burden and cost of these stations will increase. Furthermore, if the central station is destroyed, the whole uplink system will be stopped. Therefore, the focus of this paper is on the problem of maintaining the traffic balance.

The total tasks may decrease heavily if only the traffic balance is considered, and thus, the maximum task is one of the objects should be considered. The encoding method affects the genetic operations and the computation efficiency. Most of the studies encode the tasks one by one in a timely order. This does not make use of the special scene for satellite uplink. The encoding method in [13] is also inconvenient for cancelling conflicts. Therefore, the encoding method is one of the problems aimed in this paper.

This paper is organized as follows. The next section describes the visibility between the navigation constellation and the ground station. Section 3 gives the mathematical statement in the algorithm. The detailed operation of the immune genetic algorithm is described in section 4. The numerical results and analysis are presented in section 5. The final section presents our conclusions.

## 2 Visibility between navigation satellites and ground stations

Most navigation constellations consist of MEO satellites located in the orbit around 20,000 km. Because of the complex high–speed relative motion of the satellites, it is necessary to introduce the visibilities between navigation satellites and ground stations before scheduling. For example, the BeiDou constellation includes 5 GEO satellites, 27 MEO satellites, and 3 inclined geosynchronous orbit (IGSO) satellites. The altitude of the GEO satellites is 35,786 km, and they are located at 58.75°E, 80°E, 110.5°E, 140°E, and 160°E. The altitude of the MEO satellites is 21,528 km, the inclination is 55°, and they form a 24/3/1 walker constellation with 3 backup satellites. The altitude of the IGSO satellites is 35,786 km and the inclination is 55°. Then the visibilities of arbitrary MEO satellites to stations Beijing, Kashi, and Sanya can be simulated using the satellite tool kit (STK).

**Fig 1:**
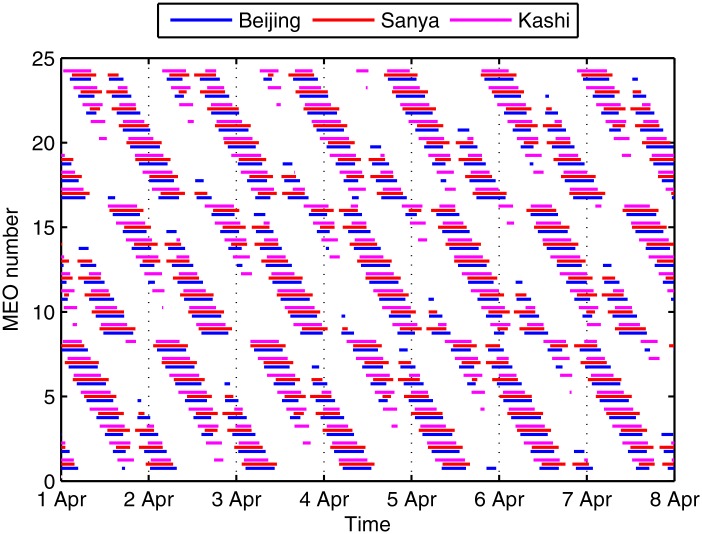
The visibilities of BeiDou MEO satellites and ground stations.

Fig 1 shows the access between the BeiDou MEO satellites and ground stations in a week, where the time step is 60 s, the elevation angle belongs to (5°, 90°), MEO*ij* is the *j*th (1,2,3,4,5,6,7,8) satellite in the *i*th (1,2,3) plane. We can see that the visibilities are similar and periodic, which is caused by the periodic motion of satellites and the Earth. At any certain moment, the satellites viewed by Beijing and Kashi are from 11 to 15 and viewed by Sanya are from 12 to 17. In addition, the longest continuous visible time is approximately 593 min, and the shortest continuous visible time is approximately 5 min. Moreover, the disconnected time is as long as 1,181 min. Therefore, scheduling the dozens of moving satellite to several ground stations is clearly a challenging work.

## 3 Mathematical statement

Mathematical Modeling is essential for complex system [25]. Consider, a set of satellites and a set of ground stations, then the model of the MuRRSP problem can be expressed as

MuRRS={S,G,T,W} (1)

Where *S* is the set of satellites, *G* is the set of ground stations, *T* is the set of uplink tasks and *W* is the set of visibility windows. To elucidate the problem, a few more variables should be defined.

The inputs are

*N*, the number of satellites;

*M*, the number of ground stations;

*N*_*T*_, the number of uplink tasks;

*N*_*W*_, the number of visibility windows;

*sat*_*i*_, *i* ∈ [1, *N*], the *i*th satellite in set *S*;

*gnd*_*i*_, *i* ∈ [1, *M*], the *i*th station in set *G*;

*N*_*ij*_, the number of visibility windows from satellite *i* to ground station *j* so that the total visibility windows is $N_{W}=\sum_{i=1}^{N}\sum_{j=1}^{M}N_{ij}$;

$w_{sat_{n},gnd_{m}}^{i}$ is the *i*th visibility window from satellite *n* to ground station *m*, which subjects to $w_{sat_{n},gnd_{m}}^{i}=\left[start_{sat_{n},gnd_{m}}^{i},end_{sat_{n},gnd_{m}}^{i}\right]$.

The outputs are

$T_{sat_{n},gnd_{m}}^{i}$, the *i*th task from satellite *n* to ground *m*; $T_{sat_{n},gnd_{m}}^{i}=1$ means the task is assigned to ground *m*, otherwise, $T_{sat_{n},gnd_{m}}^{i}=0$;

The assumptions are

1. Each uplink lasts *T*_*up*_; the task will not be considered if the visibility window is shorter than *T*_*up*_. To make full use of the ground station, the long visibility window is divided into short slices, which lasts for *T*_*up*_.
2. The interval between the adjacent slices is *T*_*idle*_, and this is the shift time of different satellites.
3. There are adequate devices in each station to serve all the visible satellites, and thus the conflicts caused by the unavailability of resources are avoided.

The target of this paper is to achieve the most balance traffic between ground stations and lower the cost as much as possible. So it is a multi-objective optimization problem [26]. Therefore, two variables should be defined first: one is the balance factor, the other is the total tasks scheduled, and they are subjected to

$$\left\{\begin{array}{c} delta=max(gnum(m))-min(gnum(m))\\ N_{tot}=\text{sum}\left(gnum\left(m\right)\right) \end{array}\right.$$ (2)

Where *m* ∈ [1,*M*], $gnum\left(m\right)=\sum_{n=1}^{N}\sum_{j=1}^{N_{nm}}T_{sat_{n},gnd_{m}}^{j}$ represents the total tasks assigned to ground station *m*, *delta* is the biggest deviation of traffic in all the stations, *N*_*tot*_ is the total tasks scheduled of all the stations. Then the objective function can be formed as

$$\min(delta+1/N_{tot})$$ (3)

As seen in Eq (3), multi objects are included. Unlike the single object method, this maintains the balance traffic and at the same time maximizes the task number. Meanwhile, there are some constraints that should be added to Eq (3).

1. Each task time equals *T*_*up*_. $\forall T_{sat_{n},gnd_{m}}^{i}=1$, the corresponding visibility window is subjected to
  $$end_{sat_{n},gnd_{m}}^{i}-start_{sat_{n},gnd_{m}}^{i}=T_{up}$$ (4)
2. A satellite should not be assigned to multi ground stations at the same time. $\forall T_{sat_{n},gnd_{m1}}^{i},T_{sat_{n},gnd_{m2}}^{j}\in T$, if $T_{sat_{n},gnd_{m1}}^{i}=T_{sat_{n},gnd_{m2}}^{j}=1$, then $w_{sat_{n},gnd_{m1}}^{i}\cap w_{sat_{n},gnd_{m2}}^{j}=\phi$, that is
  $$\left|{\text{start}}_{sat_{n},gnd_{m1}}^{i}-{\text{start}}_{sat_{n},gnd_{m2}}^{j}\right|\geq T_{up}+T_{idle}$$ (5)

We should note that the visibility window of the same satellite to different stations might be different.

## 4 The proposed immune genetic algorithm

**Fig 2:**
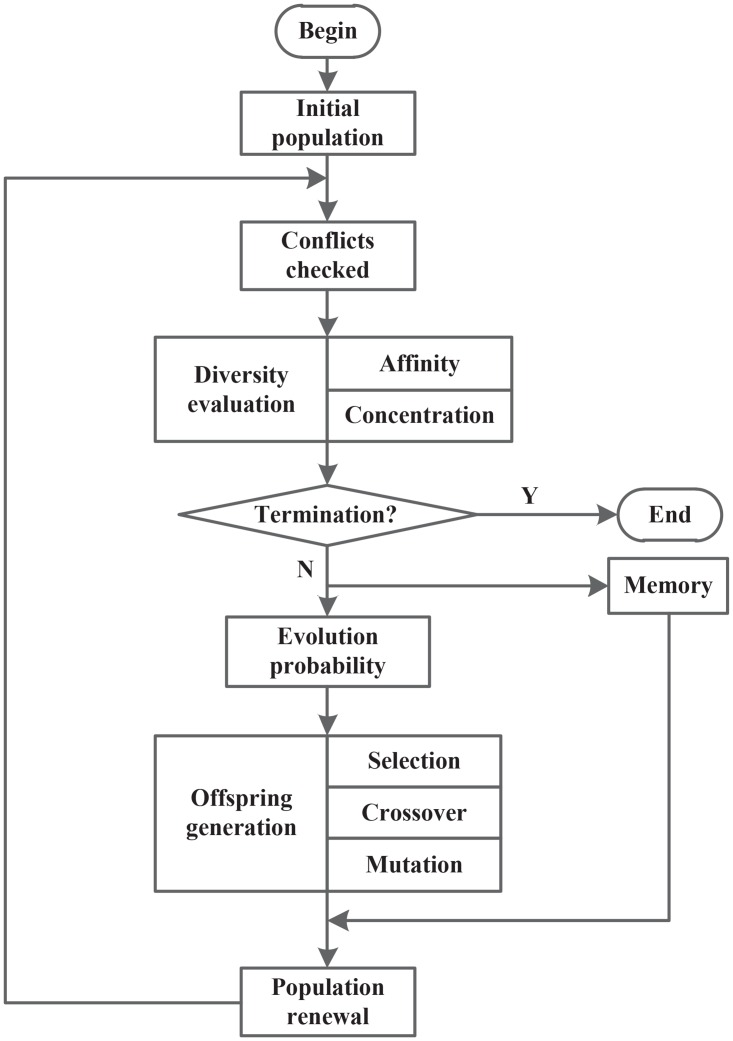
The flow of IGA.

The immune genetic algorithm is a modification of the simple genetic algorithm, thus their processes are similar. The major differences lie in individual evaluation and memory library. The diversity evaluation of individual can help avoid dropping in the local optimal solution. The detail processing flow is shown in Fig 2.

### 4.1 Encoding

In this paper, we propose a satellite–ground hierarchical encoding method. The first hierarchy is ordered by satellite.

$$Chrom=\left[G_{1},G_{2},\ldots ,G_{N}\right]$$ (6)

Where *G*_*n*_ means the uplink tasks of the *n*th satellite, the second hierarchy in a satellite can be expressed as

$$G_{n}=\left[\begin{array}{cccc} T_{sat_{n},gnd_{1}}^{1} & T_{sat_{n},gnd_{1}}^{2} & \cdots & T_{sat_{n},gnd_{1}}^{N_{n1}}\\ T_{sat_{n},gnd_{2}}^{1} & T_{sat_{n},gnd_{2}}^{2} & \cdots & T_{sat_{n},gnd_{2}}^{N_{n2}}\\ \vdots & \vdots & \ddots & \vdots \\ T_{sat_{n},gnd_{M}}^{1} & T_{sat_{n},gnd_{M}}^{2} & \cdots & T_{sat_{n},gnd_{M}}^{N_{nM}} \end{array}\right]$$ (7)

**Fig 3:**
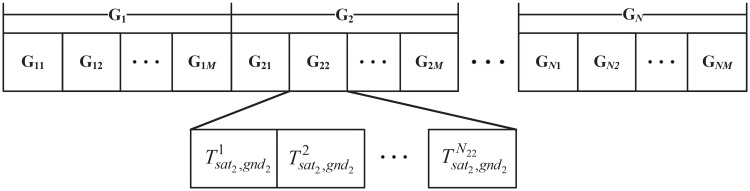
Structure of the chromosome.

Where *N*_*nm*_ is the task number of satellite *n* to ground station *m*, each row as $G_{nm}=\left[T_{sat_{n},gnd_{m}}^{1},T_{sat_{n},gnd_{m}}^{2},\ldots ,T_{sat_{n},gnd_{m}}^{N_{nm}}\right]$, represents the total task from satellite *n* to ground station *m*. If we arrange the tasks by satellites and ground stations, then the chromosome can be shown as Fig 3.

As seen in Fig 3, the chromosome is formed by *N* gene series, which contains *M* genes and each gene includes *N*_*nm*_ locus. According to the definitions in section 3, the length of the chromosome equals the number of visibility windows. If $T_{sat_{n},gnd_{m}}^{i}=1$, it implies that the task is selected, otherwise, $T_{sat_{n},gnd_{m}}^{i}=0$. This encoding chromosome is suitable for genetic operations. Meanwhile, the hierarchical code is efficient, since conflicts can be only checked in the same hierarchy.

### 4.2 Initial antibody population

The population is composed of antibodies, which are encoded as Fig 3. The IGA need initial solutions and search the optimal solution based on them. For simplicity, we initial each antibody by uniformly distributed pseudorandom integers between 0 and 1. Although there might be some conflicts, they will be removed later.

### 4.3 Conflicts checked

After genetic operations, especially the crossover, the antibody is significantly transformed. There may be plenty of conflicts in the offspring antibody according to Eq (5). Since we assumed that there are adequate devices in the ground station, the conflicts emerged when a satellite shifted between two different stations. We cancel the conflicts randomly.

### 4.4 Antibody diversity

The diversity evaluation includes two parts: affinity and concentration. The affinity is defined based on the objective function

$$A=\frac{1}{delta+1/N_{tot}}$$ (8)

As seen from Eq (8), the affinity increases with balance traffic and larger tasks. Furthermore, *A* < 1, when *delta* ≠ 0 and *A* = *N*_*tot*_ > >1, when *delta* = 0. Thus, there is an obvious step in the affinity, which may lead the solution to quickly converge to the local optimal solution. Therefore, a proportion factor should better be added to make the affinity smoother. On the other hand, if the deviation is strictly constrained to zero, the evolution speed will be harmed as well. The fuzzy method is a good way to enhance the search probability and it is introduced to the affinity function [27]. Then Eq (8) can be changed to

$$A=\frac{1}{\prod_{j=1}^{M}\left(delta-j+1\right)+k/N_{tot}}$$ (9)

Antibody concentration is used to evaluate the similarity of population. In this paper we define the concentration as

$$C\left(i\right)=\frac{1}{N_{pop}}\sum_{j=1}^{N_{pop}}x(i,j)$$ (10)

Where *i*, *j* is antibody index, *N*_*pop*_ is population scale, *x*(*i*, *j*) is the similarity flag, which is defined as

$$x\left(i,j\right)=\left\{\begin{array}{c} 1\;\;\;,\;\;\;\;s\left(i,j\right)>s_{0}\\ 0\;\;,\;\;\;\;others \end{array}\right.$$ (11)

Where *s*_0_ is a constant, which means the similarity threshold, *s*(*i*, *j*) = 2*N*_*s*_/(*L*_*i*_ + *L*_*j*_), where *L*_*i*_ and *L*_*j*_ are the length of selected tasks in antibody *i* and *j*, and *N*_*s*_ is the same alleles in the two antibodies.

### 4.5 Evolution probability

Compared to the standard genetic algorithm, the immune genetic algorithm considers the impact of affinity and concentration together. The evolution probability is decided by Eq (12)

$$P\left(i\right)=\frac{\alpha A}{\sum_{j=1}^{N_{pop}}A}+\frac{(1-\alpha )/C}{\sum_{j=1}^{N_{pop}}\frac{1}{C}}$$ (12)

Where *α* is a constant to evaluate the diversity of antibody. From Eq (12) we can conclude that IGA encourages the high affinity antibody, while it controls the high concentration one. In addition, this can help the population escape premature convergence.

### 4.6 Offspring generation

Genetic operation is the main way to generate offspring. In this paper, roulette selection, two–point crossover and single point mutation are used in IGA.

**Fig 4:**
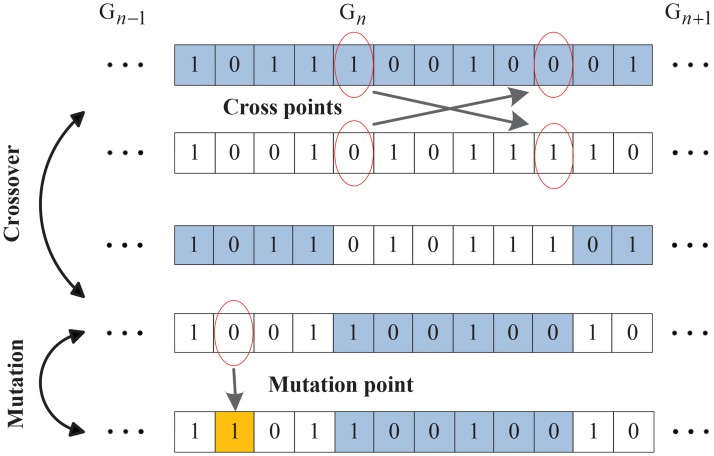
Genetic operation on antibodies.

Fig 4 shows that the two selected antibodies exchange their alleles within the two random points in crossover and turn to the opposite at the random bit in mutation.

### 4.7 Population renewal

The best several antibodies in last generation are stored in memory library, and the offspring are generated by genetic operations. To speed up the convergence of IGA, we put the excellent parents and offspring together and choose the best antibodies as the next population.

## 5 Numerical results

### 5.1 Simulation conditions

In this section, simulation and experimental results are performed to verify the feasibility and performance of the proposed method. All the experiments are implemented by M–files in MATLAB R2010a. The navigation system used for the test is the MEO satellites in BeiDou as described in section 2. Other parameters are listed in Table 1.

**Table 1:** Parameters in simulation.

Parameters	Value	Comments
Constellation	24 satellites	MEOs in BeiDou constellation
Ground station	3	Beijing, Sanya, Kashi
Elevation angle	(5°,90°)	Angle of antennas in station
Duration	24 hours	Optimal period
*T*_*up*_	30 min	Uplink time of a task
*T*_*idle*_	5 min	Shift time between the tasks

Besides, the parameters used in IGA are important for the performance and they are listed in Table 2.

**Table 2:** Paremeters in IGA.

Parameters	Value	Parameters	Value
Population scale	100	Memory scale	10
Crossover probability	0.5	Mutation probability	0.5
Selection strategy	roulette	Similarity threshold	0.8
Diversity factor	0.95	Smooth factor	400

### 5.2 Results and analysis

This section provides numerical results to examine the behavior of the IGA designed in section 4. The first set of results consider the convergence performance of the proposed method under a certain parameter. The second set of results consider the optimization of the proposed method. The next set of results show the performance under different scale of problems. The fourth set of results give the contrast effects between the multi objects and single object, thus demonstrates the cost of task number for the balance traffic object. The last set of results show the contrast between the IGA and the SGA, which reflects the advantage of the proposed method.

#### 5.2.1 Performance of the proposed method

Many factors affect the performance of immune genetic algorithm such as initial population, genetic probability, selection strategy, etc. We present the results in 20 independent runs together.

**Fig 5:**
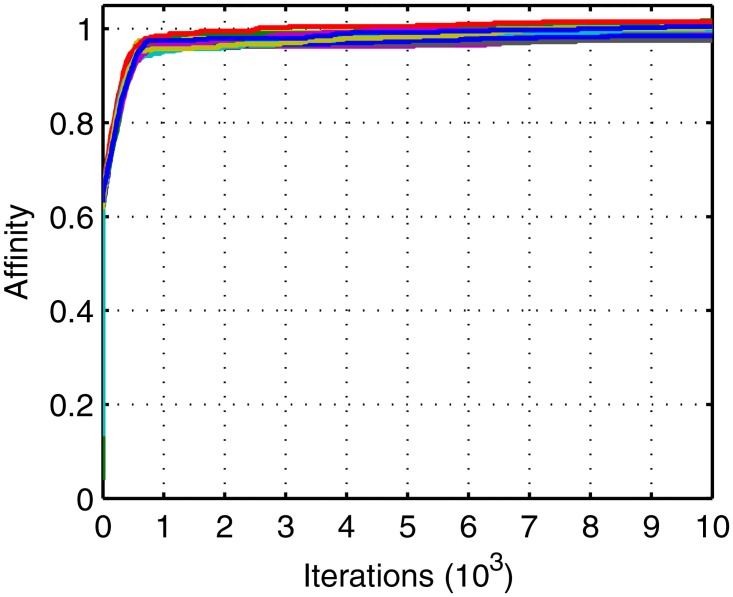
Evolution curves of affinity.

**Fig 6:**
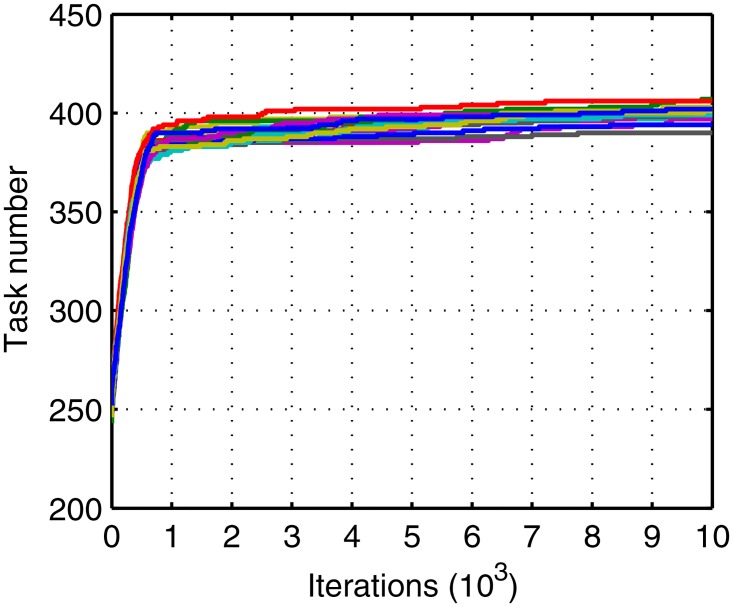
Evolution curves of maximum task.

**Fig 7:**
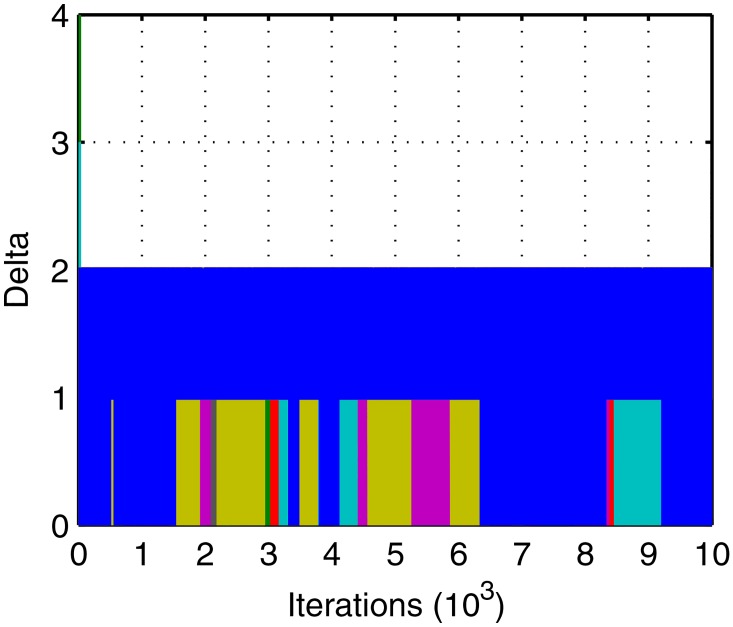
Evolution curves of traffic deviation.

Figs 5–7 shows the evolution curves of the proposed IGA. Their rapid convergence to the stable status exhibits its good performance. Figs 6 and 7 exhibit contradictory behavior and it demonstrates that the total number becomes larger and the balance is high, which proves the correctness of the method. The initial traffic is near balance because of the random generation of population but later the traffic can keep balance due to the optimal target in Eq (9). A short deviation in the tasks will cause a big suppression in affinity so that the balance is dominant in evolution. Meanwhile, when the deviation is shorter than two there exist many patterns whose total tasks are different. Of these, the larger ones have the bigger probability to appear in the offspring. Thus, the total task increases with time.

**Fig 8:**
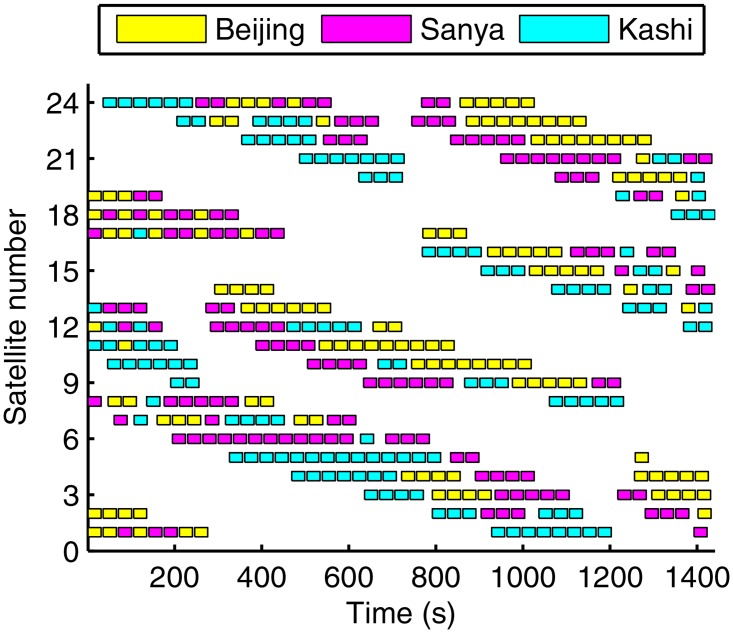
The scheduling results in a run.

Fig 8 provides the scheduling results in a run, whose task is the largest in the 20 runs. Each rectangle filled with different colors denotes a served task, and different colors represent different ground stations. As seen in Fig 8, the ground stations and the satellites are evenly placed so that we can draw a conclusion that the proposed method can find a balance solution. We should note the solution is feasible but might not be optimal, and the computation cost is relatively higher than that in traditional algorithm such as heuristic algorithm.

#### 5.2.2 The function of fuzzy deviation

The antibody affinity is defined by Eq (9) in section 4. However, a more direct definition maybe as

$$A=\frac{1}{delta+k/N_{tot}}$$ (13)

The figures below give the mean affinity and mean task of the two different objects in the 20 runs.

**Fig 9:**
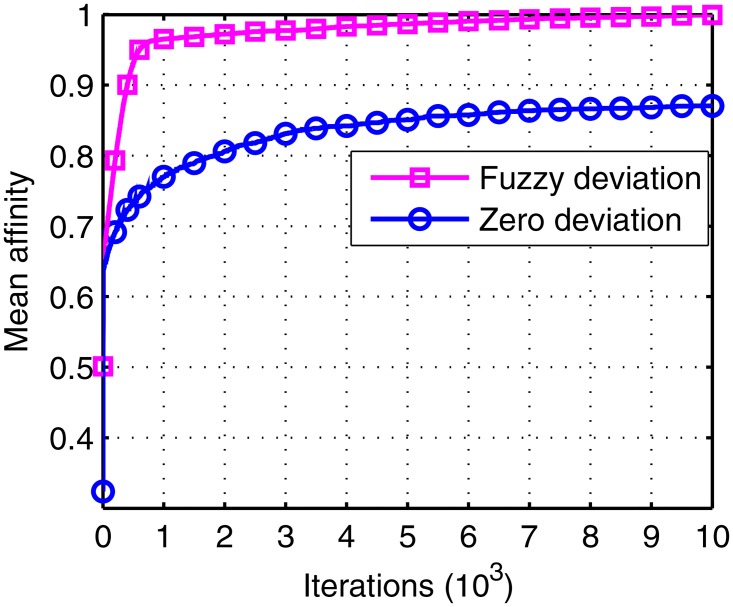
The affinity under zero deviation and under fuzzy deviation.

**Fig 10:**
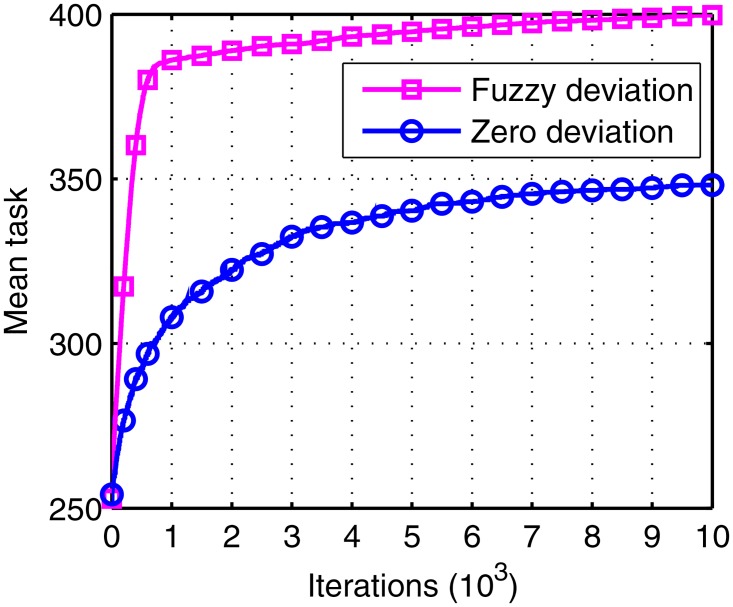
The task number under zero deviation and under fuzzy deviation.

All the parameters used for Figs 9 and 10 remain the same except the deviation measurement. From these figures, we can observe that the slope of fuzzy deviation is sharper than that in the zero deviation, which implies that the convergence speed increased. The reason for this is that the zero deviation criteria are so strict that only the same traffic can be selected. Therefore, the tasks in zero deviation only increase with the times of ground station, which is harder, while the fuzzy one accumulates the tasks little by little. Thus, the fuzzy deviation we proposed is superior.

#### 5.2.3 Comparison with different scale of problems

**Fig 11:**
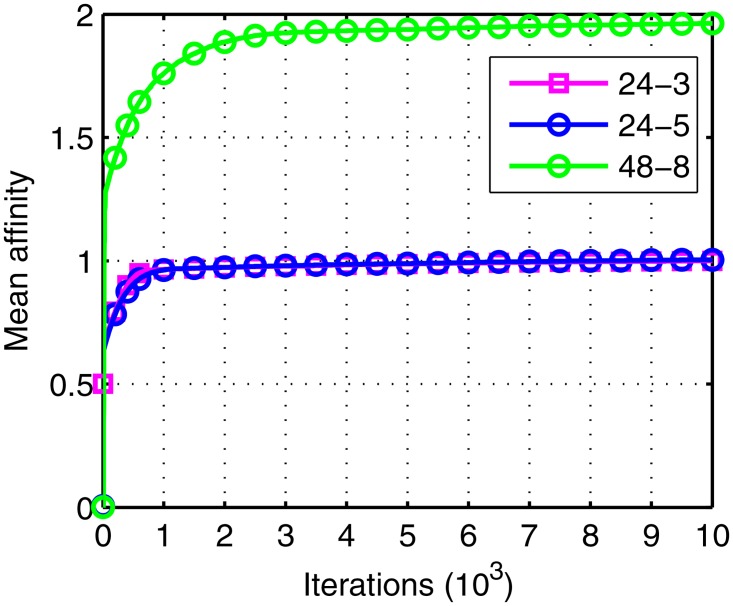
The mean affinity of different scale problems.

In order to test the flexibility of IGA, the algorithm is applied in different scale of problems. A basic GPS constellation (24 satellites) is added and its corresponding parameters are configured as those in [28]. Meanwhile, more stations including Jiamusi, Wuhan, Chengdu, Lasa and Xian are added. The mean affinity of different scenes in the 20 runs are shown in Fig 11.

As shown in Fig 11, 24–3 represents BeiDou constellation with Beijing, Sanya and Kashi; 24–5 represents BeiDou constellation with Beijing, Sanya, Kashi, Jiamusi and Wuhan; 48–8 means BeiDou and GPS constellations with all the stations. All the curves converge to the steady status, although the scale of problems are different. The affinity of 24–3 and 24–5 are almost the same while the affinity of 48–8 is approximately twice as them. Because the affinity is decided by *N*_*tot*_ when *delta* = 0 according to Eq (9). Besides, *N*_*tot*_ is decided by satellite number. Therefore, the IGA is suitable for different scale of problems.

#### 5.2.4 Comparison with the maximum task object

In order to test the effectiveness of the optimal object, a contrast is done with the maximum task object under the same IGA.

**Fig 12:**
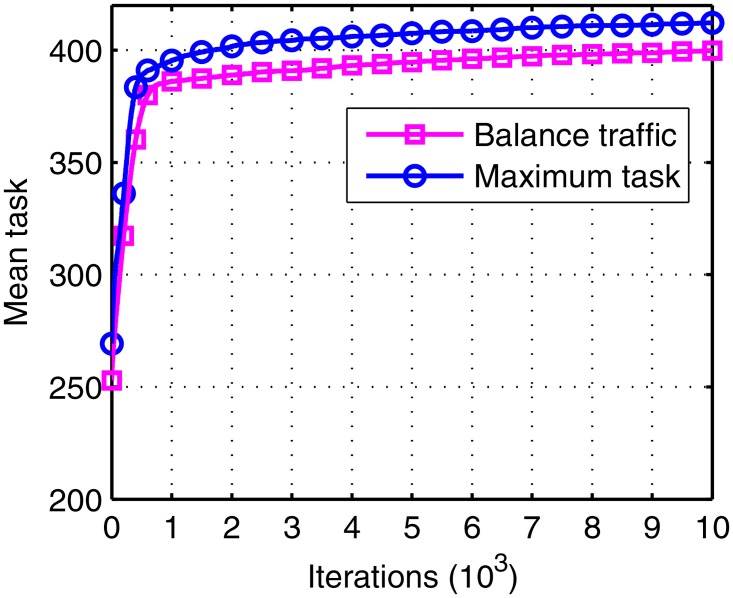
The mean task of balance traffic object and the maximum task object.

**Fig 13:**
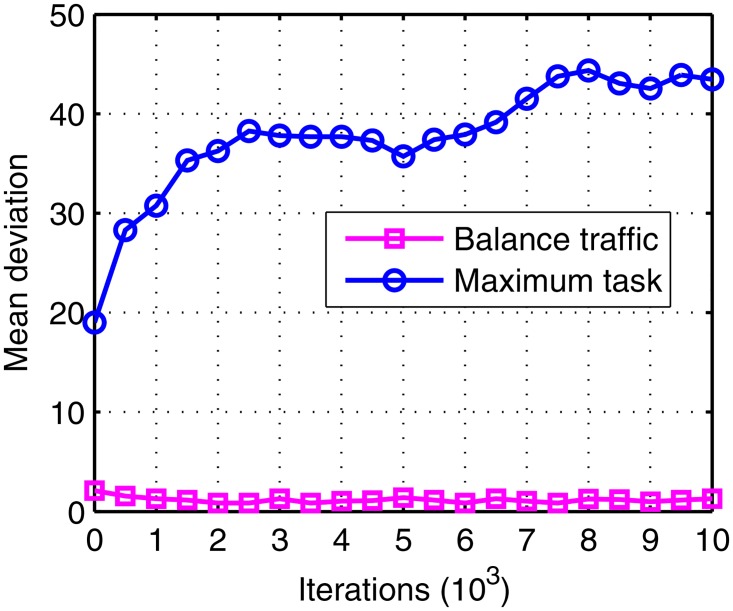
The mean deviation of balance traffic object and the maximum task object.

The mean task and mean deviation of the 20 runs are shown in Figs 12 and 13. The total tasks in the maximum task object are larger, while the deviations are more unbalance as well. The reasons include two aspects: first is the convergence of the algorithm, since the balance object is more complex than the maximum object; second is the visibility window, as the visible window may be unbalance or even cannot view the satellite at all in the scheduling time. However, the second reason can be nullified as in this paper the MEO satellite is visible to all the stations as shown in Fig 1. In detail, the total task loses about 2.98% (from 412.1 to 399.8) in the balance traffic object.

**Fig 14:**
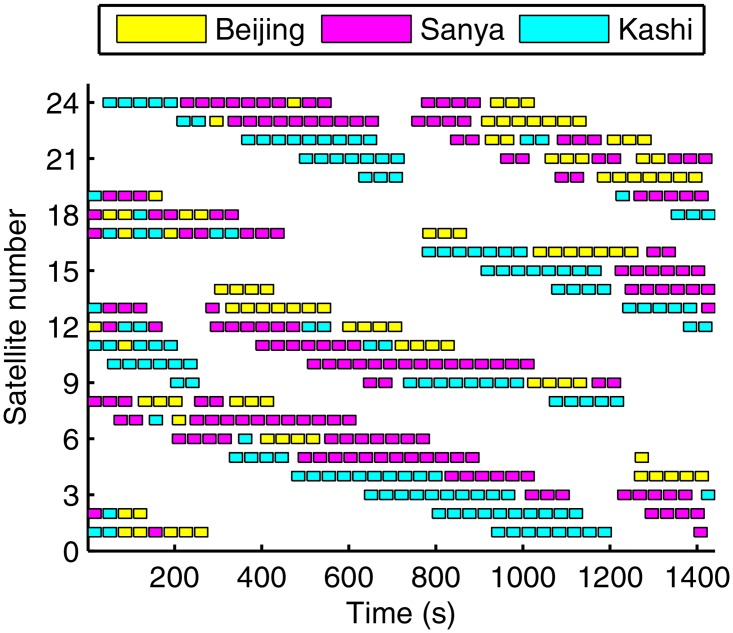
The scheduling results in a run.

Fig 14 illustrates scheduling results in a run, whose task is the largest in the 20 runs. The tasks served by Beijing, Sanya, and Kaishi are 95, 175, and 146, respectively. The traffic in Sanya is almost twice of that in Beijing, which is harmful to make use of all stations. Furthermore, the uplink capability will decrease by 42.07% if Sanya breaks down.

#### 5.2.5 Comparison with the SGA

**Fig 15:**
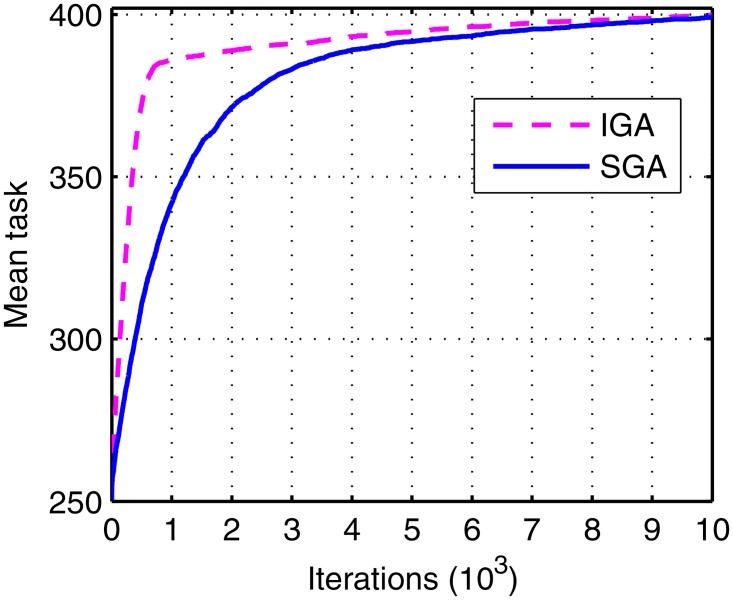
The mean tasks of IGA and SGA.

To test the advantage of IGA, the contrast is performed with SGA. They are tested under the same conditions and the mean tasks in the 20 runs are shown in Fig 15.

The curve obtained using SGA lags behind the one obtained using IGA. The differences come from the diversity evaluation and memory library modified from the SGA. The diversity evaluation, which combines affinity and concentration, can prevent the high concentration antibody accumulation at the beginning of evolution, to escape from the premature convergence of population. On the other hand, the memory library can recognize and memorize the excellent antibody, which can fasten the evolution process.

## 6 Conclusion

In this paper, we proposed a multi–objective immune genetic algorithm based on a multi–hierarchical encoding method. Several conclusions can be drawn from the derivation and numerical simulation. First, the immune genetic algorithm was successfully applied to the uplink problem of navigation constellation, and was found to be a suitable method for different scale of problems. Next, the fuzzy deviation toleration method is capable of speeding up the convergence. Moreover, the traffic balance target can be achieved with a negligible task loss (approximately 2.98%) using the proposed method. Finally, the performance of IGA is superior compared to that of the SGA.
